# Identification of Behavior Change Techniques and Engagement Strategies to Design a Smartphone App to Reduce Alcohol Consumption Using a Formal Consensus Method

**DOI:** 10.2196/mhealth.3895

**Published:** 2015-06-29

**Authors:** Claire Garnett, David Crane, Robert West, Jamie Brown, Susan Michie

**Affiliations:** ^1^ Department of Clinical, Educational and Health Psychology University College London London United Kingdom; ^2^ Cancer Research UK Health Behaviour Research Centre University College London London United Kingdom

**Keywords:** smartphone apps, alcohol consumption, consensus, Delphi technique, behavior change techniques

## Abstract

**Background:**

Digital interventions to reduce excessive alcohol consumption have the potential to have a broader reach and be more cost-effective than traditional brief interventions. However, there is not yet strong evidence for their ability to engage users or their effectiveness.

**Objective:**

This study aimed to identify the behavior change techniques (BCTs) and engagement strategies most worthy of further study by inclusion in a smartphone app to reduce alcohol consumption, using formal expert consensus methods.

**Methods:**

The first phase of the study consisted of a Delphi exercise with three rounds. It was conducted with 7 international experts in the field of alcohol and/or behavior change. In the first round, experts identified BCTs most likely to be effective at reducing alcohol consumption and strategies most likely to engage users with an app; these were rated in the second round; and those rated as effective by at least four out of seven participants were ranked in the third round. The rankings were analyzed using Kendall’s W coefficient of concordance, which indicates consensus between participants. The second phase consisted of a new, independent group of experts (n=43) ranking the BCTs that were identified in the first phase. The correlation between the rankings of the two groups was assessed using Spearman’s rank correlation coefficient.

**Results:**

Twelve BCTs were identified as likely to be effective. There was moderate agreement among the experts over their ranking (W=.465, χ^2^_11_=35.8, *P*<.001) and the BCTs receiving the highest mean rankings were self-monitoring, goal-setting, action planning, and feedback in relation to goals. There was a significant correlation between the ranking of the BCTs by the group of experts who identified them and a second independent group of experts (Spearman’s rho=.690, *P*=.01). Seventeen responses were generated for strategies likely to engage users. There was moderate agreement among experts on the ranking of these engagement strategies (W=.563, χ^2^_15_=59.2, *P*<.001) and those with the highest mean rankings were ease of use, design – aesthetic, feedback, function, design – ability to change design to suit own preferences, tailored information, and unique smartphone features.

**Conclusions:**

The BCTs with greatest potential to include in a smartphone app to reduce alcohol consumption were judged by experts to be self-monitoring, goal-setting, action planning, and feedback in relation to goals. The strategies most likely to engage users were ease of use, design, tailoring of design and information, and unique smartphone features.

## Introduction

Excessive alcohol consumption is a serious problem for population health [1,2]. Brief interventions to address this are time limited interventions delivered by health care workers targeting heavier drinkers and can be effective at reducing alcohol consumption [3]. There are substantial barriers to their delivery such as lack of time, training, and financial resources. These barriers can perhaps be avoided by delivering an intervention via a digital platform. While digital interventions have not been found to be as effective as face-to-face brief interventions [4], they may be more effective than no intervention [4-13], and have the advantage of being cost effective, avoid the stigma associated with help-seeking in person [10], and have greater reach than traditional health services. Smartphone applications or ‘apps’ have the additional advantage of being with the individual almost all of the time, which offers the potential to engage users in real time and in their everyday situations. Apps also have the ability to sense and report locations and events (in conjunction with calendar function) to provide moment-to-moment support when it is needed unlike traditional interventions. Despite a large number of apps to reduce excessive alcohol consumption in the general population, none, to our knowledge, have been rigorously evaluated. There has been a recent trial of an app on the related issue of recovery from alcoholism [14] that showed a reduction in the number of risky drinking days and therefore of probable benefit to patients in continuing care for alcohol dependence.

Reviews of digital interventions (not apps) suggest they can be effective, but there is substantial heterogeneity between different interventions [4,7,8,11,12]. Moreover, interventions have many components and their evaluations have rarely specified content in a way that would allow identification of the components responsible for the variation (e.g. [4,8,11,12]). A reliable method for specifying content and evaluating the effectiveness of complex behavior change interventions is to identify behavior change techniques (BCTs) [15]. BCTs are defined as the smallest, observable, replicable components with the potential to bring about change in behavior [16].

In order for an alcohol reduction app to be effective, it must be engaging for users, thus allowing them to be exposed to its active components. It is well established that a large proportion of users of digital interventions in health trials do not maintain engagement [17]. This degree of attrition undermines the potential of apps to be effective, and generalizable evaluation is made difficult when a large proportion of users cannot be recontacted due to disengagement with the intervention [18]. Engagement in Web-based interventions is increased by use of prompts [19-21], peer support [19], counselor support [19], and the combination of tailored communication with the use of reminders and incentives [22]. However, these have only been examined in the context of websites and there is a need to identify the most effective strategies for engagement with apps.

In sum, there is not yet an established evidence base to draw on to inform the selection of BCTs or engagement strategies in developing apps aimed at reducing alcohol consumption amongst the general population. In areas of research where there is a lack of, inconsistent, or contradictory scientific evidence, formal consensus methods have been used to guide action [23,24]. This study used a formal consensus building methodology with a small group of world-class experts in the field of alcohol and/or behavior change to identify intervention components judged to be the ‘best bets’ to reduce alcohol consumption (in general and in the context of an app) and to maintain engagement with an app, and then compared the original expert group’s ranking of intervention components with a new, broader expert review.

This study addressed the following research questions:

1. What BCTs do experts in the field of alcohol research agree are most likely to be effective in general and when delivered by an app?
2. What engagement strategies do experts believe are most likely to be effective initially and over time?

## Methods

### First Phase: 3-Round Consensus Exercise

#### Study Design

A Delphi-style methodology was used to generate consensus among experts about what intervention components are likely to be the most effective at reducing alcohol consumption, and what strategies are most likely to improve engagement with an app. Experts were asked to generate a list of ‘best bet’ intervention components and engagement strategies which were subsequently rated and ranked.

The Delphi method of generating consensus was selected as a formal, systematic and reproducible method of arriving at a consensus. It was conducted anonymously to avoid biases produced by perceived authority, persuasion or bandwagon effects [23,25].

##### Participants

Seven international academic experts (six male) were purposively identified from a range of scientific networks and backgrounds (health psychology, biological psychology, developmental psychopathology and addiction research) on the basis of their knowledge of the alcohol literature, and/or experience of designing or delivering behavior change interventions. Seven participants are considered sufficient for reliable group judgment [24,25]. None of the experts were identified based on any user experience expertise. The authorship team used their experience to judge the suitability of invited experts. Once the experts were identified, each was formally approached by an email invitation. All the experts who were approached agreed to take part. Experts were from the UK (n=6) and the Netherlands (n=1). Six were professors and one was a senior research fellow.

##### Measures

###### Round 1:

Participants were asked to provide between three and five responses to each of three questions:

1. What intervention components do you believe would be the best bets for helping people reduce their alcohol consumption?
2. What intervention components do you believe would be the best bets for helping people to reduce their alcohol consumption when delivered by a smartphone app?
3. What do you think are the best strategies or techniques for maintaining engagement with an app aiming to help people reduce their alcohol consumption?

Each question was preceded by the statement: “Please answer the following questions based on your knowledge of the research literature, relevant theory and your clinical experience. Please also provide the reason behind your choice.” For question 2, participants were given the option to indicate that their answers were the same as for question 1.

###### Round 2:

Participants were provided with an alphabetical list of the responses generated in the first round for each of the questions. They were instructed “Please rate your agreement with each of these techniques for the three different questions on the five-point Likert scales provided”. The scale ranged from 1 (*strongly disagree*), 2 (*disagree*), 3 (*neither agree nor disagree*), 4 (*agree*) to 5 (*strongly agree*). Participants were given the option to make comments on their rating.

###### Round 3:

The *n* responses were listed alphabetically with the mean agreement rating and rationale provided for each response. Participants were asked to rank the *n* responses from 1 (*most likely to be a best bet*) to *n* (*least likely to be a best bet*) for each of the questions. At this stage, participants were only asked to rank responses about which there had been broad agreement in the previous round, defined as a minimum of four out of seven of the participants agreeing (i.e., rating of 4 or above) that the technique was likely to be either effective or engaging (depending upon the question) [23]. The reason for removing responses about which there was little agreement was to improve responding by minimizing the time required to complete the survey [23]. There was the option to make any final comments at this point.

##### Procedure

This study was conducted using the online survey tool Qualtrics. A link to the survey for each of the three rounds was emailed to the participants and they were given between one and two weeks to complete it. Non-responders were sent reminders until all participants had completed each round. Participants provided informed consent.

##### Analysis

###### Round 1:

For each question, similar responses were summarized and combined. For question 1, a BCT was selected from one of two taxonomies [15,26] to describe each response for the intervention components, where appropriate. The summarizing, combining and coding of responses was conducted by CG & SM.

###### Round 2:

The mean, standard deviation (SD), and mode of the agreement ratings for each response to each of the three questions were calculated.

###### Round 3:

The final rankings were analyzed by calculating Kendall’s W coefficient of concordance [27], which measures the extent to which judges agree on their rankings of items. The value of W ranges from 0 (indicating no consensus) to 1 (indicating perfect consensus) between participants. A value of .1 corresponds to very weak agreement, .3 to weak agreement, .5 to moderate agreement, .7 to strong agreement and .9 to unusually strong agreement [28]. The Kendall’s W statistic uses the χ^2^test to test the independence of the ranking of the components.

### Second Phase: External Validation

#### Study design

The intervention components generated and ranked in the first phase of the study were also ranked by a second group of experts in the field of alcohol.

#### Participants

Assistant and Senior Editors (n=179) from the journal Addiction were invited to take part in the study if they believed they had a sufficiently informed ‘opinion on interventions that might help people who drink more alcohol than is good for them to reduce or quit’. This invitation yielded 43 participants.

#### Measures

Participants were asked to rank from 1 (highest) to 12 (lowest), the value of 12 responses generated in the first phase of the study by the original group of experts, in response to the question “What intervention components do you believe would be the best bets for helping people reduce their alcohol consumption?”

#### Procedure

An email was circulated to all the assistant and senior editors at the journal of Addiction with an alphabetical list of the “best bet” intervention component responses. If they wished to take part in the study, they were asked to reply (via email) with a ranking for each of the intervention components. Participants were given one week to reply before the study closed.

#### Analysis

The correlation between the rankings of the original and the new independent group of experts was assessed using Spearman’s rank correlation coefficient. The new rankings were also analyzed using Kendall’s W coefficient of concordance [27] to assess the extent to which this second group agreed with each other.

## Results

### First Phase: 3-Round Consensus Exercise

In response to the question of what intervention components are likely to be the most effective at reducing alcohol consumption, 24 responses were recorded in round 1. Eighteen of these responses were similar to at least one other, resulting in 12 components (see Multimedia Appendix 1), of which 11 corresponded directly with a BCT (see Table 1). Six of the 7 participants thought that intervention components likely to be effective in general would be the same as in an app. The other participant generated one suggestion to do with the intervention modality itself and how to present the intervention in a unique way. The response was therefore included with the responses to the question regarding engagement strategies.

Four of the 12 components (self monitoring, goal setting, action planning, and feedback in relation to goals) had a mean ranking score greater than the average rank (6 out of 12) and the lowest mean agreement rating for these four BCTs was 4.3 (see Table 1). Overall the original group of experts displayed moderate agreement (Kendall’s W=.465) in their ranking of intervention components (χ^2^_(11)=_35.77, *P*<.001).

**Table:** Responses generated by the expert group on effective behaviour change techniques to reduce alcohol consumption.^a^

Responses generated	Equivalent BCTs	Agreement rating^b^			Ranking score^c^	
		Mean (SD)	Mode	Agree : Disagree^d^	Mean (SD)	Mode
Self monitoring	Self monitoring of behavior^e^	4.6 (.54)	5	7:0	2.4 (1.81)	1
Goal setting	Goal setting (behavior)^e^	4.7 (.049)	5	7:0	2.6 (1.51)	1, 2
Action planning	Action planning^e^	4.3 (.49)	4	7:0	4.3 (.95)	4
Feedback in relation to goals	Provide feedback on performance^f^	4.6 (.54)	5	7:0	4.43 (2.70)	3
Behavior substitution	Behavior substitution^f^	4.1 (.38)	4	7:0	6.3 (2.06)	5, 7
Environmental triggers and drivers	Advise on environmental restructuring^f^	3.9 (.69)	4	5:2	7.3 (4.07)	2, 9
Provide information	Provide information on consequences of excessive alcohol consumption & reducing excessive alcohol consumption^f^	4.0 (.58)	4	6:1	7.4 (4.47)	12
Feedback in relation to people	Provide normative information about others’ behavior and experiences^f^	4.0 (.58)	4	6:1	8.4 (1.90)	7
Motivational interviewing	Conduct motivational interviewing^f^	3.9 (1.07)	4	5:2	8.4 (3.41)	12
Inhibition training		3.6 (.54)	4	4:3	8.4 (3.51)	10
Reward	Provide rewards contingent on successfully reducing excessive alcohol consumption^f^	3.9 (.69)	4	5:2	8.9 (2.12)	11
Habit reversal	Habit reversal^f^	3.4 (.79)	4	4:3	9.1 (1.68)	10

^a^Responses ordered in terms of mean ranking score (from round 3).

^b^Agreement rating (1: *strongly disagree*, 5: *strongly agree*).

^c^Ranking score (1: *highest*, 12: *lowest*).

^d^Agree:Disagree (ratio of (*agree/strongly agree*): (*neither/disagree/strongly disagree*) used as inclusion criteria for round 3.

^e^BCTs as referred to in the 93-item BCT Taxonomy v1 [15]

^f^BCTs as referred to in the 42-item excessive alcohol reduction specific taxonomy [26]

Of the 20 engagement strategies generated, six were similar to at least one other and thus were combined, which resulted in 17 unique strategies (see Multimedia Appendix 2 for the rationale for each of the 17 responses). Seven strategies (ease of use, design aesthetic, feedback, function, ability to change design to suit own preferences, tailored information and unique smartphone features) had a mean ranking score greater than average rank (8 out of 16) and the lowest mean agreement rating for these strategies was 3.6 (see Table 2). Overall the experts showed a moderate degree of consensus in their ranking of the strategies (Kendall’s W=.563, χ^2^_15_=59.2, *P*<.001).

**Table 2:** Responses generated by the expert group on engagement strategies.^a^

Responses	Agreement rating^b^			Ranking score^c^	
	Mean (SD)	Mode	Agree:Disagree^d^	Mean (SD)	Mode
Ease of use	4.9 (.38)	5	7:0	1.4 (.79)	1
Design – aesthetic	4.6 (.54)	5	7:0	3.1 (1.57)	2, 5
Feedback	4.6 (.54)	5	7:0	3.9 (1.68)	4
Function	4.0 (.82)	4	5:2	6.6 (3.60)	11
Design – ability to change design to suit own preferences	3.6 (.79)	4	5:2	6.9 (4.74)	3
Tailored information	4.3 (.76)	4, 5	6:1	7.9 (3.39)	6, 7
Unique smartphone features	4.4 (.54)	4	7:0	7.9 (5.79)	6
Prompts	4.1 (.38)	4	7:0	8.4 (2.44)	8
Graded tasks	4.0 (.82)	4	5:2	8.7 (3.50)	12
Gamification	4.1 (.69)	4	6:1	8.9 (5.30)	10
Social comparison	3.9 (.69)	4	5:2	10.4 (3.36)	9
Reward type Novelty	4.0 (.82)	4	5:2	11.6 (2.23)	12
Reward type Games	3.7 (.49)	4	5:2	11.9 (2.97)	11, 15
Reward type Positive messages	4.0 (.58)	4	6:1	12.1 (2.79)	8, 10, 11, 12, 13, 15, 16
Reward type Financial	3.6 (.98)	4	4:3	12.3 (1.98)	13
Social connectivity	4.0 (.58)	4	6:1	14.1 (1.95)	15, 16
Reward type- cue signaling reward^e^	3.4 (.98)	3	3:4	-	

^a^Responses ordered in terms of mean ranking score (from round 3).

^b^Agreement rating (1: *strongly disagree*, 5: *strongly agree*).

^c^Ranking score (1: *highest*, 16: *lowest*).

^d^Agree:Disagree (ratio of (*agree/strongly agree*): (*neither/disagree/strongly disagree*) used as inclusion criteria for round 3.

^e^This response was not included in round 3 because there was not substantive agreement that it would be an effective engagement strategy in round 2 (defined as a minimum of 4 out of 7 of the participants agreeing (i.e., rating of 4 or above) that the technique was likely to be engaging).

### Second Phase: External Validation

The ranking of the BCTs by the original group was validated by an independent group of experts: there was a significant correlation between their two rankings (see Table 3; ρ=.69, *P*=.01). Table 3 shows the ranking by the independent group of experts of the intervention components generated and agreed by the original group. There was modest but significant agreement amongst the broader group of experts (Kendall’s W=.320, χ^2^_11_=151.52, *P*<.001).

**Table 3:** Comparison between rankings of phase 1 expert group and larger expert group of effective behavior change techniques for alcohol use reduction.^a^

Responses	Phase 1 experts	Phase 2 experts
	N=7	N=43
	Mean Rank (SD)	Mean Rank (SD)
Self monitoring	2.4 (1.81)	3.4 (2.88)
Goal setting	2.6 (1.51)	3.8 (3.00)
Action planning	4.3 (.95)	6.4 (2.72)
Feedback in relation to goals	4.4 (2.70)	4.1 (2.28)
Behavior substitution	6.3 (2.06)	7.6 (2.51)
Environmental triggers and drivers	7.3 (4.07)	5.1 (2.72)
Provide information	7.4 (4.47)	9.5 (2.87)
Feedback in relation to people	8.4 (1.90)	7.4 (3.27)
Motivational interviewing	8.4 (3.41)	7.2 (2.82)
Inhibition training	8.4 (3.51)	8.8 (2.15)
Reward	8.9 (2.12)	6.8 (3.44)
Habit reversal	9.1 (1.68)	7.9 (2.69)

^a^Responses ordered in terms of mean ranking score for the original experts (from round 3)

## Discussion

BCTs of self monitoring, goal setting, action planning, and feedback in relation to goals were ranked most likely to be effective for reducing alcohol use by a group of international experts in the field of alcohol or behavior change or both. This finding was validated by a larger independent group of alcohol experts. None of the experts thought that the BCTs likely to be effective in general would differ from those in an app, though one participant suggested presenting information in a way that was unique to an app. The most highly ranked engagement strategies were ease of use, design-aesthetic, feedback, function, design-ability to change design to suit own preferences, tailored information and unique smartphone features.

There is empirical evidence for the effectiveness of some of the BCTs identified in this study for reducing excessive alcohol consumption. Self monitoring has been found to be effective in brief interventions [26], and is also used in a number of apps to reduce alcohol consumption [29] though none of these have been evaluated. The BCT ‘feedback in relation to people’ is often referred to as normative feedback in the alcohol behavior change literature. There is evidence to suggest that this BCT may have a small effect by several different modes of delivery: face-to-face [30], via phone [31], mailed [32,33] and via digital platforms [30,34,35]. However, this research is often limited to college and university students [30,32,34,35]. The highest priority engagement strategies of prompts, social connectivity and tailored information have all been shown to result in increased use of Web-based interventions [19-22].

The use of a Delphi approach to selecting intervention components is clearly not guaranteed to result in the best choices, but on a priori grounds it seems preferable to the more usual practice of drawing on expertise and interest within a single research team. It may have been that no consensus would be achieved so, while the level of agreement within each group of experts was modest, the fact that the aggregate rankings of the two expert groups showed a high level of concordance was reassuring that the study tapped into a shared perspective on the existing evidence.

It is possible that the results of the Delphi exercise could have been biased by choosing an expert group with similar backgrounds to those of the research team. Therefore, the use of a second group of experts to validate the rankings provided important support for this not being the case. The journal Addiction has a very large pool of international experts on its editorial team and arguably includes most of the leading researchers in the field covering a wide range of expertise. The question regarding user engagement was included for exploratory purposes. As shown in this study, experts in the academic field of research did not identify any BCTs as being effective for an app compared with a traditional intervention. This may be because they are not aware of the additional functions an app can provide in terms of a behavior change intervention. Future research is planned to compare the views of experts in the relevant academic field with that of user experience experts to see if there are any discrepancies between these groups and if so, how their opinions differ.

The results of this study will be used to inform the building of a prototype app that will be evaluated in a field experiment. Following the principle of optimization [36] each component will be included in a full form or minimal form using a factorial design so that its effect can be assessed. The findings should also be useful to other research teams considering developing and evaluating apps in this area.

## Appendix

Multimedia Appendix 1 Intervention components generated by the experts in the first round.

Multimedia Appendix 2 Engagement strategies generated by the experts in the first round.

## Abbreviations

BCTs: behavior change techniques
NIHR SPHR: National Institute of Health Research, School for Public Health Research
SSA: UK Society for the Study of Addiction
UKCTAS: UK Centre for Tobacco and Alcohol Studies
