# Supplementary material for: Identification of Behavior Change Techniques and Engagement Strategies to Design a Smartphone App to Reduce Alcohol Consumption Using a Formal Consensus Method
Source: JMIR Mhealth Uhealth. 2015 Jun 29;3(2):e73. doi: 10.2196/mhealth.3895 (PMC4526967; doi:10.2196/mhealth.3895)
Supplement: Multimedia Appendix 1 [file mhealth_v3i2e73_app1.pdf]

Appendix 1

Round 1 open-ended responses and rationale generated by the experts to the following question:

What intervention components do you believe would be the best bets for helping people reduce their alcohol consumption?

1. Action planning

- Plans for how to avoid specific triggers for drinking or cope with the motivation to drink when it occurs
- Self-monitoring is more effective when combined with action planning

2. Behaviour substitution

- Prompt substitution of the unwanted behaviour with a wanted or neutral behaviour
- E.g. instead of having a birthday party at a bar, doing some other activity, such as paintballing

3. Environmental triggers and drivers

- Identify specific triggers and drivers that generate the urge/want/need to drink
- E.g. pressure to attend pub after work regularly

4. Feedback in relation to goals

- Related to the one or more goals set and any cumulative goals
- Needs to give warnings as get near the limit for session, day, week, etc
- Individualised feedback and information has more relevance and salience

5. Feedback in relation to people

- Consistent overestimation of the drinking levels of peers
- Social norms/normative feedbacks approaches are effective in correcting misperceived norms to reduce alcohol consumption and alcohol-related problems

6. Goal-setting

- Set clear goals for a) session, b) day, c) week and cumulative goals
- Tailored to the needs of the client
- Meaningful and achievable goals

7. Habit reversal

- Prompt rehearsal and repetition of an alternative behaviour to replace an unwanted habitual behaviour
- E.g. have a soft drink every other round

8. Inhibition training

- Poor inhibitory control has been found to have a causal link with heavy drinking and alcohol problems
- Inhibitory control can be experimentally manipulated ('trained'), which results in reduced alcohol consumption
- Multiple training sessions may be a viable way to help people to reduce their alcohol consumption
- An example of inhibition training is approach-avoidance training: where participants are implicitly trained to avoid or approach alcohol-related stimuli

9. Monitoring

- People are generally unaware of the level at which they drink
- Important accompaniment to goal setting
- As easy and quick to implement as possible
- Options for different ways of doing it (e.g repeat last drink, catch-up on missed drinks)

10. Motivational interviewing

- Techniques involving prompting the client to provide self-motivating statements and evaluations of own behaviour to minimise resistance to change
- Proven effective in drug abuse and related problems

11. Provide information

- Raising awareness of the consequences of drinking different amounts of alcohol might prompt behaviour change
- Brief information (when given by expert/authorative figures like doctors) can be very effective to curb problem drinking and related problems
- This could include information on the alcohol related risks for different demographic groups

12. Reward

- Provide rewards contingent on successfully reducing excessive alcohol use/abstaining
