# Supplementary material for: Identification of Behavior Change Techniques and Engagement Strategies to Design a Smartphone App to Reduce Alcohol Consumption Using a Formal Consensus Method
Source: JMIR Mhealth Uhealth. 2015 Jun 29;3(2):e73. doi: 10.2196/mhealth.3895 (PMC4526967; doi:10.2196/mhealth.3895)
Supplement: Multimedia Appendix 2 [file mhealth_v3i2e73_app2.pdf]

Appendix 2

Round 1 open-ended responses and rationale generated by the experts to the following question:

What do you think are the best strategies or techniques for maintaining engagement with an app aiming to help people reduce their alcohol consumption?

1. Design - Ability to change design to suit own preferences

- Option for changing look and feel of the app when the user wants
- Tailor app to different aesthetic tastes

2. Design - Aesthetic

- Needs to look good
- Attractive and simple design

3. Ease of use

- Needs to feel good/easy to use
- Intuitive user interface

4. Feedback

- Immediate, informative feedback
- Illustrates progress towards a goal
- Show if there is any improvement in task performance

5. Function

- Range of different functions
- Capture different processes (e.g. monitoring, associations)

6. Gamification

- Typical game elements (e.g. incremental challenges, monitoring progress, giving rewards) are used for different purposes
- Makes the intervention more persuasive
- Strategies should be tailored to fit different age groups

7. Graded tasks

- Motivating when you achieve a higher level
- Can help with self-efficacy

8. Prompts

- Can enhance interaction

9. Reward type - Cue signalling reward
10. Reward type - Financial
11. Reward type - Games
12. Reward type - Novelty

- E.g. accessing a new element/section of the app

13. Reward type - Positive messages
14. Social - Comparison

- Option for comparing self with others
- Anonymous unless permission granted

15. Social - Connectivity

- Social support to achieve a certain goal
- Works well in other health behaviour apps e.g. weight loss

16. Tailored information

- Provide cumulative personalised information about user in relation to others

17. Unique smartphone features

- Use of features/functions only available on a smartphone
- For example, prompts/reminders based on location or time
